# Medical Notes

**Published:** 1849-03

**Authors:** 


### Medical Notes. By an Assistant Surgeon, U. S. Navy.

[Reported for the Medical Examiner.]

The medical visitor to Brazil cannot fail to remark the large number of persons that are affected with enlargement of the scrotum. Excessive hypertrophy of that part may constantly be met with, forming a mass distressing by its weight and position. It may be found weighing many pounds, reaching below the knees, obstructing locomotion, and requiring artificial supports of various kinds. In one individual in the city of Rio de Janeiro it was known to have reached such a size as to have required to be carried in a sort of barrow in advance of the body.

The disease appears to be confined to the scrotum and consists in an hypertrophy of its tissues. The testicles are not involved, but are found of normal appearance in the upper portion of the mass. Hydrocele is however frequently co-existent. The nature of the affection is variously regarded by the native physicians: many think it of imported origin, caused by intercourse with African negroes labouring under the "yaws;" others that it arises from offensive habits, and want of cleanliness amongst the natives.

It would appear in fact to be an endemic disease, due to some peculiarity of the soil and climate, and allied to chronic erysipelatous inflammation "of *periodic* type." Persons of correct moral habits are often found the subjects of the disease, and a variety of it is met with among females of the upper class in whom the labia and adjoining parts are the seat of the hypertrophied condition. It is not improbable that the disease may be of malarious origin, having a well marked paroxysmal character. An attack is most generally ushered in by symptoms resembling those of intermittent fever. There is the preceding mal-aise, a well marked chill, and hot stage, and the crisis of the paroxysm is marked by an augmentation of the tissue of the scrotum, and the patient is at the same time relieved of all feelings of indisposition. New and repeated attacks take place at various intervals, and with successive deposits.

The scrotum finally loses its rugose appearance, the integuments of the penis are called upon to support and invest the tumour, that organ itself disappearing in the mass, its position being indicated only by its urethral orifice.

The disease has been variously treated: extirpation, and removal by the knife, have been resorted to, but the practitioners of the country have much hesitation in operating, as the practice cannot be said to be generally successful. In all cases the existence of hydrocele should be ascertained, and the treatment should commence by the removal of the fluid, great relief to the integuments being afforded in the diminution of the bulk of the mass; the relaxation of the tissue can also be promoted by steaming the part, and by local depletion by leeches, scarifying, &c.

Simple, medicated, and vapour baths, by their soothing and cleansing influence, and blisters and setons by acting as local drains, are of great service. The system at the same time should be maintained in a healthy tone, attention being directed to the state of the secretions and activity of the emunctories, whose agency is of peculiar value in the removal of abnormal deposites. Constitutional and alterative remedies, iodine and its salts, sulphur and the antimonials, the infusion, decoction and local application of indigenous plants supposed to have a specific effect on the surface, enter into the treatment.

The arsenical preparations are those which are found to have most influence in arresting and removing the disease, and their efficacy may be as much ascribed to their *anti-periodic power* as to their alterative action; quinine, which in the same view would appear to be indicated, has not been used in this affection; its employment, however, has been suggested, and if found successful would assist in determining the pathology.

The disease in the majority of cases has been found to resist all treatment.

In females, the labia and adjoining hypertrophied integuments have been removed with success.

